# Strength training under hypoxic conditions

**DOI:** 10.14814/phy2.12227

**Published:** 2015-01-19

**Authors:** Jesús Álvarez‐Herms, Sonia Julià‐Sánchez, Michael Hamlin, Ginés Viscor

**Affiliations:** Departament de Fisiologia‐Immunologia, Facultat de Biologia, Universitat de Barcelona, Barcelona, Spain (J.H., S.J., G.V.); Department of Tourism, Sport and Society, Lincoln University, New Zealand (M.H.)

## Introduction

Recently, Kon et al. (^5^) published in this journal an interesting paper entitled “Effects of systemic hypoxia on human muscular adaptations to resistance exercise training “ which determined the effect of 8 weeks of resistance training (5 sets of 10 reps at 70% of 1 RM of free weight bench‐press and bilateral leg‐press using a weight‐stack machine) under hypoxia (HRT, 14.4% O_2_) or normoxia, on muscle cross‐sectorial area, 1 RM, muscular endurance and markers of mitochondrial biogenesis and angiogenesis. Their conclusion suggested two important findings: (1) an increased muscular endurance and, (2) the promotion of angiogenesis in skeletal muscle in the hypoxic‐trained subjects compared to the normoxic‐trained subjects. However, in our opinion, a number of the comments in the Kon et al. (^5^) publication need clarification, moreover, the author's conclusions may only partially explain the benefits of resistance training in hypoxia for endurance athletes.

In the discussion section, the author's state: “For the first time, we demonstrate a significant resistance‐training‐induced increase in muscular endurance in the HRT group”. This comment fails to take into consideration previously reported, improved endurance capacity after a strength‐endurance‐training program performed in hypobaric (Álvarez‐Herms et al. ^1^) and normobaric hypoxia (Manimmanakorn et al. ^6^).

Despite reporting similar change in endurance capacity to the Álvarez‐Herms et al. (^2^) study the protocol designed by Kon et al. (^5^) used a workload equivalent to 70% of 1 RM that has traditionally been used to promote skeletal muscle sarcoplasmic hypertrophy. Such hypertrophy results in enhanced body mass (Paavolainen et al. ^7^) as occurred in the Kon et al. (^5^) study (*P *< 0.05), and is perhaps not the most exercise specific method to improve the muscular endurance capacity in athletes. In this regard, Paavolainen et al. (^7^) demonstrated that during relatively short‐training periods of some weeks, improvements of endurance performance with resistance exercise was obtained primarily with explosive strength and neural adaptations without observable muscle hypertrophy. In our study (Álvarez‐Herms et al. ^2^), we designed the training program to combine resistance and explosive strength exercises in order to improve neural adaptation without subsequent muscle hypertrophy which may be detrimental to endurance athletes that must carry their body weight over long distances. We therefore suggest that for endurance athletes that want enhanced muscular endurance performance without the increased body mass that comes with muscle hypertrophy, short explosive training under hypoxia is more suitable.

Kon et al. (^5^) observed an increase in angiogensis (indicated by increased plasma VEGF concentration and capillary‐to‐fiber ratio) after the strength training in hypoxic compared to normoxic conditions. Such change would indicate that strength training (at the lower end of resistance intensity for strength improvements, 70% 1 RM) under hypoxic conditions may improve the ability of the muscles to extract oxygen from the blood and may be a useful alternative to traditional strength‐endurance training. However, Kon et al. (^5^) tested recreational athletes and it remains to be seen whether such adaptation also occurs in highly trained elite athletes.

From a practical point of view, resistance exercise performed in hypoxia could have two important points of interest for athletes: (1) it provides a higher physiological, physical, and mental stimulus compared to the same training performed in normoxia and, (2) unlike aerobic exercise under hypoxia which frequently requires athletes to travel to altitude facilities and train for long hours, anaerobic intermittent training in hypoxia may give similar performance benefits for considerably less resource”. We believe training intensity is a vitally important factor during hypoxic exercise and has a large bearing on whether adaptation and subsequent performance improvement occur or not. When the work intensity is high enough adaptation and performance improvement occur (Rusko et al. ^9^), but when work intensity is insufficient the corresponding physiological stimulus is too low resulting in less adaptation and decreased performance (Truijens et al. ^10^). In this regard, recent investigations are proving that short programs of high‐intensity exercise in hypoxia improve the anaerobic work capacity primarily due to physiological adjustments such as increased buffering capacity (Faiss et al. ^4^) or enhanced glycolityc enzyme activity (Puype et al. ^8^). At the same time, because the exposure to hypoxia is minimal, individual maladaptive susceptibility that traditionally occurs during altitude camps (chronic hypoxia) or the classical intermittent hypoxic training (Living High‐Training Low) in some subjects (Chapman et al. ^3^) is less likely. We know that subjects who are susceptible to maladaptation during hypoxic training tend to reduce training quality resulting in detrimental changes to physical performance (Chapman et al. ^3^).

In conclusion, the paper of Kon et al. (^5^). reinforces the idea that resistance training combined with hypoxia (at a relatively low‐strength‐training intensity, i.e., 70% 1 RM) could be a useful way to enhance endurance capacity but it remains to be seen whether the training program used was the most appropriate method for developing endurance capacity in endurance‐based athletes.
